# A Hospitalist’s Challenge: Systemic Botulism Following a Cosmetic Injection Requiring Antitoxin Therapy and Percutaneous Endoscopic Gastrostomy (PEG)

**DOI:** 10.7759/cureus.92391

**Published:** 2025-09-15

**Authors:** Parag N Patel, FNU Kalpana, Didar Singh, Sandipkumar Patel, Kolawole K Oyewole

**Affiliations:** 1 Medicine, Gujarat Medical Education and Research Society (GMERS) Medical College and Hospital, Patan, IND; 2 Internal Medicine, Pandit Bhagwat Dayal Sharma Post Graduate Institute of Medical Sciences, Rohtak, IND; 3 Internal Medicine, Springfield Memorial Hospital, Springfield, USA

**Keywords:** botulism neurotoxin a, bulbar muscle weakness, cosmetic botulinum toxin injection, dysphagia, iatrogenic botulism

## Abstract

Botulinum toxin type A (BoNT-A) is widely used in cosmetic practice and generally considered safe, with adverse effects usually limited to transient local reactions. Rarely, systemic spread or counterfeit formulations can cause iatrogenic botulism, presenting with bulbar weakness and generalized neuromuscular dysfunction. We report the case of a 53-year-old woman who developed progressive dysphagia, bilateral ptosis, bifacial weakness, and proximal muscle weakness following cosmetic BoNT-A injections to the face and masseter muscles. Imaging and endoscopic evaluations were unremarkable, and myasthenia gravis (MG) was excluded. Despite initial partial improvement on pyridostigmine, her bulbar dysfunction worsened, necessitating administration of botulinum antitoxin and eventual percutaneous endoscopic gastrostomy (PEG) placement for nutritional support. Over subsequent weeks, she demonstrated gradual recovery, though mild ptosis persisted at discharge. This case underscores the potential for systemic botulism after cosmetic BoNT-A, particularly in the setting of unregulated or counterfeit products. Physicians must maintain a high index of suspicion in patients with recent cosmetic injections presenting with cranial or bulbar symptoms, as timely recognition and antitoxin therapy are essential to optimize outcomes and reduce morbidity.

## Introduction

Botulinum toxin (BoNT), a neurotoxic protein produced by *Clostridium botulinum*, is one of the most potent biological agents known [1]. It has become one of the most common minimally invasive cosmetic procedures since the U.S. Food and Drug Administration (FDA) approved onabotulinumtoxinA (Botox Cosmetic, AbbVie Inc., North Chicago, IL) for glabellar lines in 2002 [2]. Beyond cosmetics, BoNT type A (BoNT-A) remains an established therapy for cervical dystonia, chronic migraine, hyperhidrosis, strabismus, and other neuromuscular disorders [3,4].

Although BoNT-A is generally safe, most adverse effects are mild and localized, such as transient eyelid ptosis or injection-site edema [3]. Rarely, systemic diffusion or counterfeit products can lead to iatrogenic botulism, manifesting with dysphagia, dysarthria, ptosis, generalized weakness, and even respiratory compromise [1]. The underlying mechanism involves irreversible binding of the toxin’s light chain to presynaptic cholinergic nerve terminals, where it cleaves SNARE proteins (particularly SNAP-25), thereby blocking acetylcholine release at the neuromuscular junction. This inhibition of synaptic transmission produces the characteristic flaccid paralysis and bulbar dysfunction seen in systemic cases [5]. Recent reports highlight an increase in cosmetic-related botulism: a case from Turkey (2024) required antitoxin therapy after cosmetic Botox injection [6]; a five-patient cluster in Turkey was reported [7]; three patients in China presented with bulbar symptoms [8]; and several patients in Iran were linked to suspected counterfeit preparations [9]. Warnings now highlight the risks of adulterated and counterfeit BoNT-A products [10]. In the United States, the Centers for Disease Control and Prevention (CDC) issued a 2024 alert highlighting more than 20 suspected cases tied to counterfeit BoNT products in non-clinical settings [11]. We present a case of a middle-aged woman who developed progressive dysphagia, bilateral ptosis, and generalized weakness after cosmetic BoNT-A injection. Her course required both symptomatic therapy with pyridostigmine and definitive management with botulinum antitoxin, followed by percutaneous endoscopic gastrostomy (PEG) placement for nutritional support. For internists, botulism represents a rare but important differential in patients presenting with acute dysphagia, ptosis, or bulbar symptoms after cosmetic procedures.

## Case presentation

A 53-year-old woman with a past history of esophageal stricture, treated with dilation three years earlier, presented with progressive throat tightness and dysphagia that began a few days after injection and progressively worsened over nine days before presentation following cosmetic BoNT-A injections to the bilateral masseter muscles, forehead, and canthal regions at a medical spa. She initially sought urgent care, where computed tomography (CT) of the neck, streptococcal and mononucleosis testing, and laboratory studies were unremarkable. She received symptomatic therapy, including ketorolac (Toradol), dexamethasone, and nebulized treatment, but reported no significant improvement. Despite this, her dysphagia worsened, and she was admitted under the Internal Medicine service, with Neurology, ENT (ear, nose, and throat), and Gastroenterology consulted. Esophagogastroduodenoscopy (EGD) ruled out mechanical obstruction and other structural causes of dysphagia.

**Figure 1:**
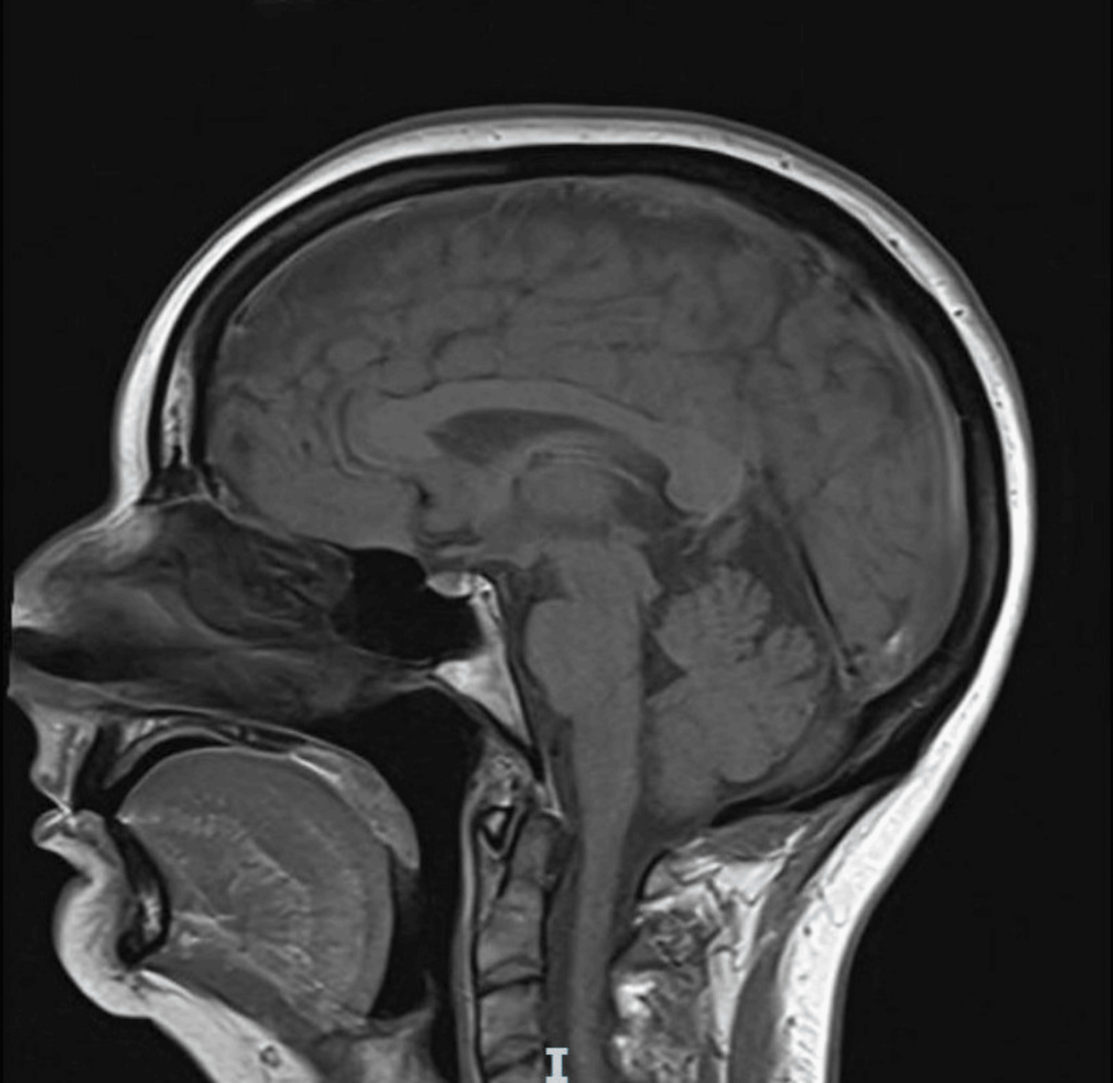
Magnetic resonance imaging (MRI) of the brain (sagittal T1-weighted image) demonstrating no acute intracranial abnormalities.

**Figure 2:**
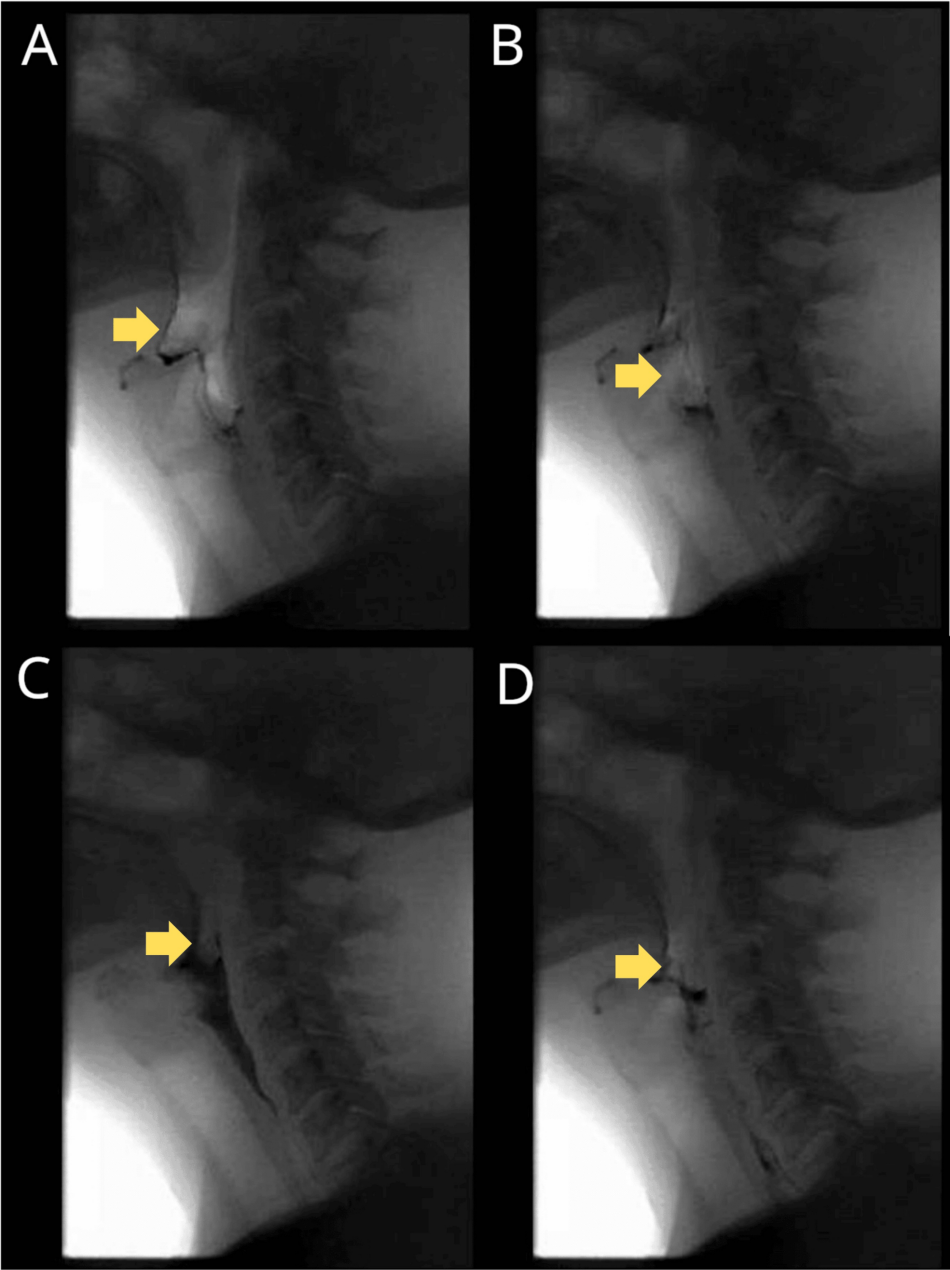
Sequential still frames from the videofluoroscopic swallow study (A) Bolus entry into the oral cavity; (B) Impaired oral propulsion with reduced tongue base retraction; (C) Residual pooling in the oropharynx without aspiration; (D) Incomplete clearance with persistent oral-phase dysfunction.

On admission (hospital day 10, post injection), the neurologic examination was notable for bilateral ptosis, facial weakness, and dysarthria, along with difficulty swallowing, without evidence of diplopia or respiratory distress. Brain MRI was unremarkable (Figure 1). Pyridostigmine was initiated at 30 mg three times daily and increased to 60 mg the following day; this was associated with partial improvement in limb strength, although bulbar dysfunction persisted. On follow-up, examination demonstrated persistent eyelid droop and dysarthria. Motor testing revealed neck flexion weakness (Medical Research Council (MRC) grade 3), proximal limb weakness (shoulders and hips MRC 4/5), but preserved distal strength. Extraocular movements showed mild adduction impairment (left greater than right). Given the ongoing decline in swallow function, Poison Control was consulted, and botulinum antitoxin was administered on hospital day 4. Subsequently, she required Keofeed placement, followed by PEG tube insertion on hospital day 10 (Figure 2), as her oral-phase dysfunction persisted and recovery timeline was uncertain, making long-term nutritional support necessary. Serology for acetylcholine receptor antibodies was negative, making new-onset myasthenia gravis (MG) less likely. Over two weeks, she improved gradually, regaining strength in facial and bulbar muscles, though residual ptosis persisted at discharge. On follow-up four weeks later, she was tolerating oral intake, had discontinued PEG feeds, and was planning PEG removal, indicating substantial neurological recovery.

## Discussion

Botulism is caused by neurotoxins produced by *Clostridium botulinum* and, less commonly, by other *Clostridium *species. Among the seven serotypes (A-G), serotypes A, B, E, and F are responsible for most human cases and represent some of the most potent biological substances known [1]. Experimental models suggest that the lethal oral dose of purified crystalline BoNT-A could be as low as 1 µg/kg [2]. In recent decades, however, iatrogenic botulism has become an important clinical entity, reflecting the widespread therapeutic and cosmetic use of BoNT-A [5]. While foodborne and wound botulism remain classical forms, iatrogenic botulism following therapeutic or cosmetic BoNT-A injections is increasingly recognized [5]. Clinically, iatrogenic botulism often begins with bulbar symptoms such as ptosis, dysphagia, and dysarthria, while sensation remains intact [1]. Its global popularity in cosmetic medicine, particularly for aesthetic facial procedures, has expanded dramatically. This rise has been accompanied by increasing reports of iatrogenic botulism, often related to high-dose injections or unlicensed formulations used in non-clinical settings [11]. Contributing factors include counterfeit distribution networks, unsafe practices such as “Botox parties,” and cross-border smuggling of unregulated products [12]. In our case, the injected dose was not available, as the patient was unable to recall the amount administered. Prior case series have reported a wide range of cosmetic doses associated with iatrogenic botulism, from as low as 100 units to greater than 300 units, often exceeding recommended cosmetic limits [5-8, 13].

In our patient, symptoms began several days after the injection and progressed over nine days before hospitalization. This timeline is consistent with prior case reports, where systemic symptoms typically emerge within two to 10 days following injection [5-8, 13]. Symptom onset shortly after injection and bulbar dysfunction mirrored the report by Richardson and Viviano (2024), where early antitoxin led to improvement [6]. In iatrogenic cases, symptoms are usually transient, paralleling the pharmacodynamic duration of the injected toxin, but can nevertheless be severe enough to require hospitalization and antitoxin. The diagnosis depends on recognizing a history of recent botulinum toxin injection in conjunction with compatible neurological findings, often necessitating hospitalization and consideration of antitoxin therapy [7]. The differential diagnosis in internal medicine is broad, encompassing Guillain-Barré syndrome, MG, brainstem stroke, and Lambert-Eaton myasthenic syndrome [1]. BoNT-A can also unmask latent MG, complicating diagnostic certainty [14]. In our case, MG was excluded on the basis of absent antibody positivity, lack of diurnal fluctuation, and the characteristic temporal association with cosmetic injections.

Prompt treatment is essential. Antitoxin, if administered within the first 48 hours of symptom onset, neutralizes circulating toxin and significantly reduces respiratory complications and mortality [1]. However, antitoxin cannot reverse neuroparalysis that has already occurred. Supportive management, including airway protection, nutritional support, and physiotherapy, remains the cornerstone of care. Pyridostigmine has been used off-label in iatrogenic botulism to improve neuromuscular transmission by inhibiting acetylcholinesterase [15,16]. In our patient, pyridostigmine provided partial benefit but did not prevent progression, necessitating antitoxin administration. PEG feeding was required to maintain adequate nutrition during the prolonged bulbar dysfunction.

Recovery typically spans weeks to months, reflecting neuronal sprouting and synaptic remodeling [17]. This aligns with outbreak data from intragastric BoNT-A cases showing protracted but complete recovery [13]. This case emphasizes several critical points. First, cosmetic BoNT-A, though widely considered safe, can rarely cause systemic botulism with life-threatening complications. Second, counterfeit or unlicensed products amplify the risk and remain a global public health concern. Third, physicians must carefully distinguish iatrogenic botulism from mimicking conditions such as MG or stroke, as early recognition enables timely antitoxin administration. Finally, medicolegal awareness is essential: only licensed products should be used, informed consent must include discussion of rare but serious complications, and unregulated distribution of BoNTs should be subject to strict enforcement.

## Conclusions

Although cosmetic BoNT-A is widely considered safe, systemic botulism remains a rare but potentially life-threatening complication. Clinicians should maintain a high index of suspicion for iatrogenic botulism in patients presenting with recent cosmetic injections and cranial or bulbar weakness. Prompt recognition, early initiation of diagnostic evaluation, and timely administration of botulinum antitoxin are critical to prevent progression and improve outcomes. The use of counterfeit or unlicensed formulations further amplifies risk, underscoring the need for stricter regulation, physician vigilance, and patient education regarding the potential hazards of non-clinical injection settings.
